# Prevalence of *Orientia tsutsugamushi*, *Anaplasma phagocytophilum* and *Leptospira interrogans* in striped field mice in Gwangju, Republic of Korea

**DOI:** 10.1371/journal.pone.0215526

**Published:** 2019-08-16

**Authors:** Mi-Seon Bang, Choon-Mee Kim, Jung Wook Park, Jae Keun Chung, Dong-Min Kim, Na Ra Yun

**Affiliations:** 1 Department of Internal Medicine, College of Medicine, Chosun University, Gwangju, Republic of Korea; 2 Premedical Science, College of Medicine, Chosun University, Gwangju, Republic of Korea; 3 Division of Infectious Disease Investigation, Health and Environment Research Institute of Gwangju City, Gwangju, Republic of Korea; Bharathidasan University, INDIA

## Abstract

This study investigated the prevalence of *Orientia tsutsugamushi*, *Anaplasma phagocytophilum*, and *Leptospira interrogans* in wild rodents through molecular detection using organ samples and through serological assay using blood samples of mice collected from two distinct sites in Gwangju Metropolitan City, Republic of Korea (ROK). A total of 47 wild rodents, identified as *Apodemus agrarius (A*. *agrarius)*, were captured from June to August 2016. The seroprevalence of antibodies against bacterial pathogens in *A*. *agrarius* sera was analyzed; 17.4% (8/46) were identified as *O*. *tsutsugamushi* through indirect immunofluorescence assay and 2.2% (1/46) were identified as *Leptospira* species through passive hemagglutination assay. Using polymerase chain reaction, the spleen, kidney and blood samples were investigated for the presence of *O*. *tsutsugamushi*, *A*. *phagocytophilum*, and *L*. *interrogans*. Out of the 47 *A*. *agrarius*, 19.1% (9/47) were positive for *A*. *phagocytophilum* and 6.4% (3/47) were positive for *L*. *interrogans*, while none were positive for *O*. *tsutsugamushi*. Four out of 46 (8.7%) blood samples, six out of 45 (13.3%) spleen samples, and one out of 47 (2.1%) kidney samples were positive for *A*. *phagocytophilum*. Three out of 47 (6.4%) kidney samples were positive for *L*. *interrogans*. The sequencing results of PCR positive samples demonstrated > 99% similarity with *A*. *phagocytophilum* and *L*. *interrogans* sequences. *A*. *phagocytophilum* was mostly detected in the spleen, whereas *L*. *interrogans* was mostly detected in the kidneys. Notably, *A*. *phagocytophilum* and *L*. *interrogans* were detected in *A*. *agrarius* living in close proximity to humans in the metropolitan suburban areas. The results of this study indicate that rodent-borne bacteria may be present in wild rodents in the metropolitan suburban areas of ROK.

## Introduction

Rodents are known carriers of zoonotic pathogenic agents that are usually transmitted to humans through direct or indirect contact [1].

Scrub typhus, caused by *Orientia tsutsugamushi*, is an infectious disease and has become one of the most prevalent human diseases in the Asia-Pacific region, including in the Republic of Korea (ROK) [2]. Since 2004, the disease has spread widely in the southwestern provinces of the country and is endemic in nature [3]. In 1995, 274 cases of scrub typhus were reported in ROK, whereas 10,365 cases were reported in 2013, indicating a 38.1-fold increase in the incidence of this disease [4].

*Anaplasma phagocytophilum* causes human granulocytic anaplasmosis (HGA). *A*. *phagocytophilum* has been detected in *Hemaphysalis longicornis (H*. *longicornis)*, *Ixodes nipponensis (I*. *nipponensis)*, and *Ixodes persulcatus (I*. *persulcatus)* ticks in ROK [5]. HGA was first identified in the United States in 1994 [6]. The incidence of anaplasmosis has also increased from 1.4 cases per million in 2000 to 6.1 cases per million in 2010 [7, 8]. The first HGA case in ROK was reported in 2013 [9].

Leptospirosis has been one of the most important endemic diseases in ROK since 1984. Wild rodents, particularly *A*. *agrarius*, are a common source of infection in ROK, especially during the harvest season in the rural areas. The prevalence of *Leptospira* infection in field rodents has been previously investigated via detection of leptospiral DNA in rodent kidneys [10, 11].

The presence of scrub typhus, anaplasmosis, and leptospirosis in ROK indicates that *O*. *tsutsugamushi*, *A*. *phagocytophilum*, and *Leptospira interrogans* have infected both rodents and humans. Therefore, it is necessary to monitor the prevalence of pathogens in animal hosts, such as wild rodents, which harbor such pathogens and vectors like ticks and mites involved in disease transmission. A limited number of studies have evaluated the prevalence of pathogens in wild rodents in the suburban areas of metropolitan cities in ROK. Especially, studies on the issue of organ tropism (i.e., prevalence rates of *O*. *tsutsugamushi*, *A*. *phagocytophilum*, and *L*. *interrogans* in different tissues) are very limited.

This study investigated the prevalence of *O*. *tsutsugamushi*, *A*. *phagocytophilum*, and *L*. *interrogans* in wild rodents through molecular detection of the pathogens in the organs of rodents collected from two distinct sites in Gwangju City, ROK. This study may broaden the understanding of health risks associated with wild rodents living in close proximity to humans.

## Materials and methods

### Collection of wild rodent samples

The rodents were captured using Sherman traps (3” × 3.5” × 9’, USA) at two sylvatic habitats located in the suburban areas of Buk-gu (35° 10′ 12″ N, 126° 54′ 36″ E) and Gwangsan-gu (35° 8′ 5″ N, 126° 47′ 40″ E) in the Gwangju metropolitan city of ROK from June to August 2016. The captured rodents were numbered sequentially. All the animals were euthanized by inhalation of 5% isoflurane in accordance with an approved animal use protocol. Blood, spleen, and kidney tissues were obtained and stored at– 80 °C for use in future experiments. All the samples were tested for the presence of *O*. *tsutsugamushi*, *A*. *phagocytophilum*, and *L*. *interrogans*.

### Ethics statement

This study was approved by the institutional review board (IRB) of Chosun University. Approval for the capturing and experiments of the animals were obtained from Chosun University Institutional Animal Care and Use Committee (CIACUC) (approval no. CIACUC2016-A0003).

### Indirect immunofluorescence assay (IFA)

The blood samples were centrifuged at 3000 rpm for 20 min, to separate the serum which was stored at 4 °C. Indirect immunofluorescence assay (IFA) and passive hemagglutination assay (PHA) were performed after 24–36 h of euthanizing the mice.

For IFA, 10 μL of sera from each *A*. *agrairus* was used in two-fold serial dilutions of 1:16 to 1:2048 in phosphate-buffered saline (PBS, pH 7.2; Welgene Inc., Korea). The diluted sera were incubated on antigen slides at 37 °C for 30 min in a humidified chamber, washed twice with sterile PBS, and again washed twice with distilled water. Fluorescein isothiocyanate (FITC)-conjugated goat anti-mouse IgG (Sigma, St. Louis, Missouri, USA) (25 μL) was added to each slide, incubated at 37 °C for 30 min in a humidified chamber, washed twice with sterile PBS and distilled water, and air-dried. A mounting medium (Sigma) was added to each slide which was covered with a cover slip. The slides were examined for specific spots under a fluorescence microscope (Carl Zeiss, Oberkochen, Germany). A cutoff titer of 1:16 was used for interpreting infection in the wild rodents. In-house antigen slides for *O*. *tsutsugamushi* were provided by the Korea Center for Disease Control (KCDC). *O*. *tsutsugamushi*-infected human serum and normal human serum provided by KCDC were used as positive and negative controls. The in-house *O*. *tsutsugamushi*-injected mouse serum and normal mouse serum were used as positive and negative controls.

### Passive hemagglutination assay (PHA)

For PHA, Genedia Lepto PHA (Green Cross, Seoul, Korea) kit reagents were used. Serum (25 μL) from each rodent was added to a 96-well plate, and diluted to 1:80 in a dissolved solution of Genedia Lepto kit. Sheep blood (75 μL) containing red blood cells sensitized to *Leptospira* species was placed in diluted serum for the agglutination assay. The Ag-Ab agglutination reaction at 1:80 dilution was found to be positive for leptospirosis.

### DNA extraction

A small piece of tissue (around ~50 mg) in 200 μL of ATL buffer of QIAamp Tissue & Blood Mini Kit (QIAGEN, Germany) was homogenized with a bead beater (BioSpec 3110BX Mini-Beadbeater-1 High-Energy Cell Disrupter). The tissue lysates were incubated at 56 °C overnight, and the genomic DNA was extracted using QIAamp Tissue & Blood Mini Kit following the manufacturer’s instructions.

### Polymerase chain reaction (PCR)

To detect the presence of *O*. *tsutsugamushi* DNA, the 16S ribosomal RNA gene (*rrs*) and 56 kDa gene (*56 kDa*) of *O*. *tsutsugamushi* were targeted. PCR was also performed using the INNOPLEX TSUTSU detection kit (cat. no. IPC10040; Intron Biotechnology, Korea). The kit contained primer sets designed to detect the 475-bp fragment of a gene encoding the 56 kDa antigen of *O*. *tsutsugamushi*. PCR was performed with 2 μL of DNA extract and 18 μL of distilled water treated with diethyl pyrocarbonate (DEPC, Gendepot, Barker, Texas, USA) in PCR premix tubes following the manufacturer’s instructions.

The heat shock protein gene (*groEL*) and ankyrin-related protein gene (*ankA*) were targeted to detect *A*. *phagocytophilum*. To detect the presence of *L*. *interrogans* DNA, RNA polymerase subunit beta (*rpoB*), outer membrane lipoprotein (*LipL32*), and DNA gyrase subunit B (*gyrB*) were targeted. All the primers used for the specific target genes, PCR conditions, and product sizes are given in Table 1. Nested PCR (nPCR) was performed in 20 μL reaction volumes using the AccuPowerR PCR PreMix (Bioneer Corp., Korea). Each PCR mixture consisted of 16 μL of distilled water, 1 μL of each primer (10 pmol/μL), and 2 μL of DNA template.

**Table 1:** Nucleotide sequences and PCR conditions for the detection of rodent-borne bacteria in the rodent tissue.

Bacteria	Target gene	Primer name	Nucleotide sequence (5′-3′)	Product size (bp)	PCR profile (°C/s)				Reference
					Denaturation	Annealing	Extension	Cycles	
*Orientia tsutsugamushi*	*rrs*	ot-16sRF1	AGGGATGATAATGACAGTACCTACAG	199	94/60	57/30	72/45	36	In this study
		ot-16sRR1	CCTCTACCATACTCTAGCCTAACAG						
	*56 kDa*	56BO-144F	YGYAGAATCTRCTCGCTTGG	1250	94/60	60/60	72/60	35	In this study
		56BO-1395R	AGCTAMCCCTRCACCAABAC						
		56BO-406F	CCWCCTCARCCTACTATRATGC	680	94/30	61/30	72/45	30	
		56BO-1088R	GCWGCTGCTRCTGCTTCTTG						
*Anaplasma* *phagocytophilum*	*groEL*	GRO607F	GAAGATGCWGTWGGWTGTACKGC	688	95/30	54/30	72/60	30	[12]
		GRO1294R	AGMGCTTCWCCTTCWACRTCYTC						
		GRO677F	ATTACTCAGAGTGCTTCTCARTG	445	95/30	57/30	72/60	30	
		GRO1121R	TGCATACCRTCAGTYTTTTCAAC						
	*ankA*	ANK-F1	GAAGAAATTACAACTCCTGAAG	705	95/30	53/30	72/60	35	[13]
		ANK-R1	CAGCCAGATGCAGTAACGTG						
		ANK-F2	TTGACCGCTGAAGCACTAAC	664	95/30	52/30	72/60	5	
		ANK-R2	ACCATTTGCTTCTTGAGGAG		95/30	54/30	72/60	25	
*Leptospira* *interrogans*	*rpoB*	rpoB-1889F	GTTCCAACATGCAACGYCAR	1649	94/60	52/60	72/60	35	In this study
		rpoB-3537R	GTTGAAGGATTCRGGRATAC						
		rpoB-2438F	TYATGCCKTGGGAAGGWTAC	1023	94/30	56/30	72/45	30	
		rpoB-3460R	GCATRTCRTCKGACTTGATG						
	*LipL32*	hap1-435F	GGGAATACGTAGAMGTTCG	1435	94/60	56/60	72/60	35	In this study
		hap1-1870R	GTTTATAGTAGGTTGAAGCTTG						
		L-hap1-217F	CCGTGATTTTCCTAACTAAGG	848	94/30	58/20	72/45	30	[14]
		L-hap1-218R	CAGATTACTTAGTCGCGTCAGA						
	*gyrB*	LeptoB2F	TGAGCCAAGAAGAAACAAGCTACA	500	94/30	59/30	72/45	35	[15]
		LeptoB504R	MATGGTTCCRCTTTCCGAAGA						

All amplifications were performed in an AB thermal cycler (Applied Biosystem, Foster City, CA, USA). The amplified products were separated by electrophoresis on a 1.2% agarose gel and stained with ethidium bromide for visualization.

### Sequencing and phylogenetic analysis

Sequencing of PCR amplicons from rodent-borne pathogens was conducted by Solgent Inc. (Daejeon, Korea). The obtained sequences were compared for similarity with the sequences deposited in the GenBank using BLAST. The gene sequences excluding the primer regions were aligned using the multisequence alignment program of Lasergene version 8 (DNASTAR, USA), and phylogenetic analysis was performed using the MEGA 6 software.

Phylogenetic trees were constructed using ClustalW of the MegAlign Program (DNASTAR, USA) based on the alignments of positive gene sequences using the neighbour-joining method. Bootstrap analysis (1,000 replicates) was performed according to the Kimura 2-parameter method. Pairwise alignments were performed with an open-gap penalty of 10 and a gap extension penalty of 0.5.

## Results

### Wild rodent collection

A total of 47 wild rodents were captured from June to August 2016 at the two trapping sites in the vicinity of Gwangju city in ROK. The wild rodents collected were: 16 mice (captured in June), 18 mice (captured in July), and 13 mice (captured in August). All the wild rodents were identified to be *A*. *agrarius w*hich were captured in fallow ground, around water, a boundary area between forest and field, and around a tomb.

### Seropositivity for *O*. *tsutsugamushi* and *L*. *interrogans* in rodent serum

The serum samples collected from 46 *A*. *agrarius* were subjected to IFA. Eight samples out of 46, (17.4%) were seropositive for *O*. *tsutsugamushi* with a cutoff titer of ≥ 1:16 for IgG. In addition, PHA was performed for the detection of antibodies against *Leptospira* species and one sample out of 46 (2.2%) serum was positive with a cutoff titer of 1:160 (Table 2).

**Table 2:** Rate of positive bacterial infections in 46 *Apodemus agrarius* rodents, as indicated by serological assays.

Name of positive sera	Seropositive	
	IFA titer for *O*. *tsutsugamushi* ^a^ (% positive rate)	PHA for *Leptospira* species ^b^ (% positive rate)
6–6	1:128	– ^c^
6–7	1:64	–
7–4	1:16	–
7–13	1:16	–
7–17	1:16	1:160
7–18	1:32	–
8–7	1:256	–
8–9	1:64	–
**No. of positive sera**	**8 (17.4)**	**1 (2.2)**

^a^ Cutoff titer of immunofluorescence assay, immunoglobulin G ≥ 1:16,

^b^ Cutoff for passive hemagglutination assay ≥ 1:80,

^c^–: negative.

### Molecular detection of bacterial pathogens in rodent tissue

The tissue samples were obtained from blood, spleen, and kidneys of 47 *A*. *agrarius*. Among the total mice samples, four blood samples out of 46 (8.7%), six spleen samples out of 45 (13.3%), and one kidney sample out of 47 (2.1%) was positive for *A*. *phagocytophilum*. Three kidney samples out of 47 (6.4%) were positive for *L*. *interrogans*, whereas and all the spleen and blood samples were negative. In contrary, all the *A*. *agrarius* tissues were negative for *O*. *tsutsugamushi* (Table 3). No co-infection was found in our study (S1 Table).

**Table 3:** Number of positive cases for *Orientia tsutsugamushi*, *Anaplasma phagocytophilum*, and *Leptospira interrogans*, among the 47 *Apodemus agrarius* rodents obtained by PCR targeting different genes.

Specimen	No. of samples	*O*. *tsutsugamushi*			*A*. *phagocytophilum*		*L*. *interrogans*		
		*rrs*^a^	TSUTSU Kit^b^	56 kDa^c^	*groEL*^d^	*ankA*^e^	*rpoB*^f^	*LipL32*^g^	*gyrB*^h^
Blood	46	0	0	0	**4**	**4**	0	0	NA^i^
Spleen	45	0	0	0	0	**6**	0	0	NA
Kidney	47	0	0	0	0	**1**	**3**	**2**	**2**
**No. of positive rodents**		0			**9 (19.1)**^j^		**3 (6.4)**		

^a^ 16S ribosomal RNA,

^b^ INNOPLEX TSUTSU detection kit for *O*. *tsutsugamushi*,

^c^ 56 kDa gene,

^d^ Heat shock protein chaperone,

^e^ Ankyrin-related protein gene,

^f^ RNase polymerase subunit beta,

^g^ Outer membrane lipoprotein,

^h^ DNA gyrase subunit B,

^i^ NA: not available,

^j^ % Positive rate.

### Sequencing and phylogenetic analysis

The genes of rodent-borne bacteria were sequenced and aligned with the sequences obtained from the GenBank database using ClustalW. The neighbor-joining tree was constructed using the Kimura 2-parameter model (1,000 bootstrap replicates) in the MEGA 6 software.

**Fig 1:**
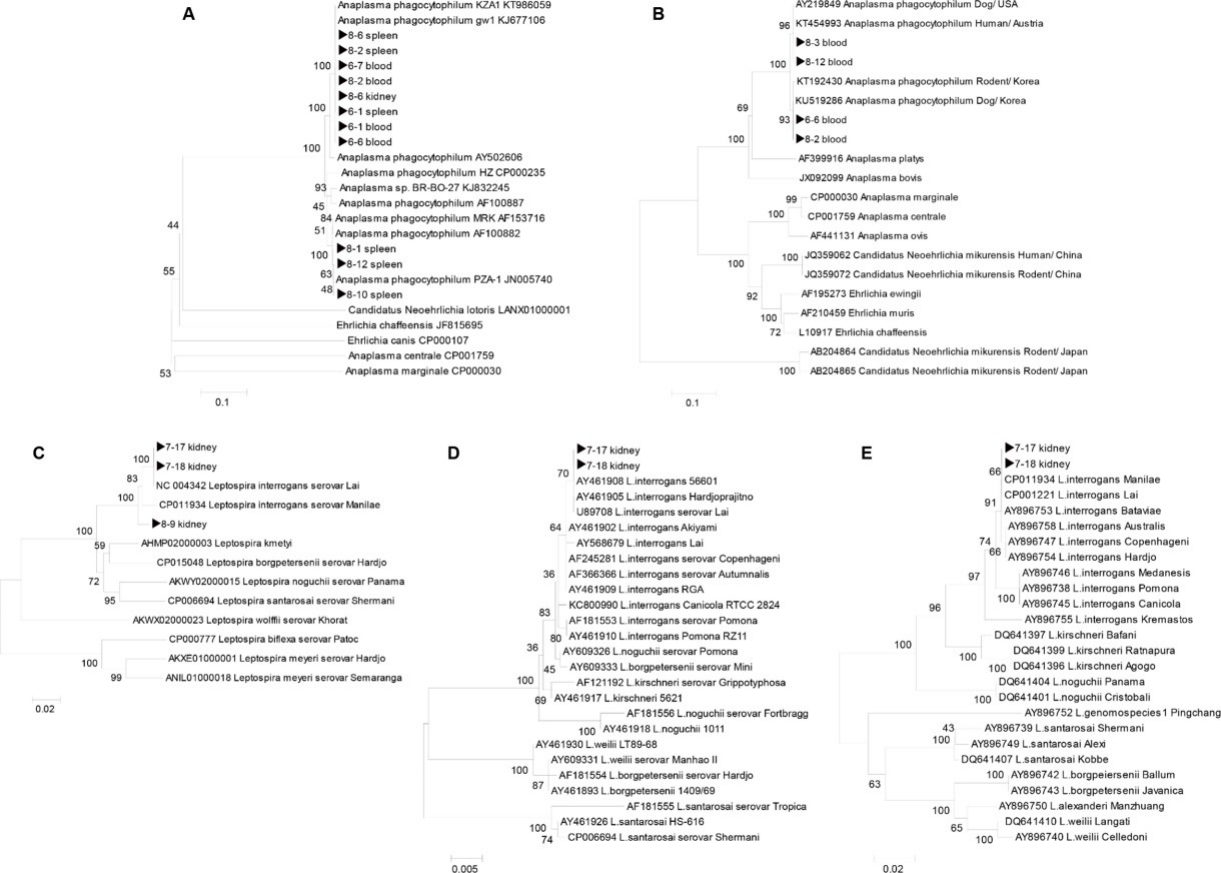
Phylogenetic trees generated based on nucleotide sequences of *A*. *phagocytophilum* and *L*. *interrogans* in tissues obtained from the *Apodemus agrarius* rodents captured in Gwangju. (A) 560 bp of *ankA*. (B) 330 bp of *groEL* gene sequences for *A*. *phagocytophilum*. (C) 890 bp of *rpoB* gene. (D) 780 bp of *LipL32* gene. (E) 400 bp of *gyrB* gene sequences for *L*. *interrogans*.

The *ankA* gene sequences collected from the rodent tissues demonstrated 99% similarity with that the of *A*. *phagocytophilum* strain isolated from humans in ROK (accession no. KJ677106 and KT986059, 100% bootstrap support, Fig 1A). The *groEL* gene sequences demonstrated 99% similarity with the *A*. *phagocytophilum* strain isolated from the rodents and dogs in ROK (accession no. KT192430 and KU519286, 93% bootstrap support, Fig 1B), and with *A*. *phagocytophilum* strain isolated from humans in Austria (accession no. KT454993, 96% bootstrap support, Fig 1B).

The *rpoB* gene sequences collected from the rodent tissues showed 99–100% similarities with *L*. *interrogans* serovar *lai* isolated from China (accession no. NC004342, 100% bootstrap support, Fig 1C), while the other samples exhibited 99–100% similarities with *L*. *interrogans* serovar *manilae* isolated from the mouse kidneys in Japan (accession no. CP011934, 100% bootstrap support, Fig 1C). The *LipL32* gene sequences demonstrated 100% similarities with *L*. *interrogans* serovar *lai* isolated from sewage in China (accession no. AY461908) and with *L*. *interrogans* serovar *hardjoprajitno* isolated from humans in Indonesia (accession no. AY461905, 70% bootstrap support, Fig 1D). The *gyrB* gene sequences demonstrated 100% similarities with *L*. *interrogans* serovar *lai* isolated from sewage in China (accession no. CP001221) and with *L*. *interrogans* serovar *manilae* isolated from mouse kidneys in Japan (accession no. CP011934, 66% bootstrap support, Fig 1E).

## Discussion

Scrub typhus, which is caused by *O*. *tsutsugamushi*, is transmitted to humans by the bites of chigger mites (*Trombiculidae*) [16]. The incidence of scrub typhus is influenced by multiple factors, such as the behavior and population density of *Trombiculidae* and wild rodents. Human activity may also influence the infection rates [17, 18]. *A*. *agrarius* is a dominant wild rodent, which harbors chigger mites carrying *O*. *tsutsugamushi* [19]. In this study, *O*. *tsutsugamushi* was not detected in 47 *A*. *agrarius* through PCR. Eight out of 46 (17.4%) sera were found to be seropositive through IFA with a cutoff titer of 1:16 for IgG. The gold standard for serological detection of scrub typhus is IFA [20] however, it lacks standardization and the use of variable cutoff titers has shown lab-wise variability in the results [21]. The usage varied significantly across different geographical areas where scrub typhus is endemic and the cutoff ranged from 1:10 to 1:400 [21]. Our IFA and PCR results did not corroborate because the time of wild rodents being infected was unclear. Kim, et al. [22] observed that IgG antibody titer increases abruptly over the first 2 weeks and reaches its peak at about 4 weeks in scrub typhus patients. The molecular techniques, such as PCR provide the highest sensitivity and specificity for the detection of scrub typhus, especially in the early period of infection, due to the specificity of primers and low detection limits [23–27]. Thus, although the antibody reaction was positive, molecular detection was not achieved in the positive mice. It may be preferable to perform both molecular and serological assays for precisely detecting the pathogens.

*A*. *phagocytophilum* has long been recognized as an animal and human pathogen and is one of the major concern to public health [28]. In previous studies, serological and molecular evidence indicated that *A*. *phagocytophilum* has spread to humans, rodents, and ticks in many Asian countries, including Korea, China, and Japan [29–32]. Chae et al. [5], have confirmed that ticks, rodents, and shrews near the Demilitarized Zone (DMZ) in Korea were positive for *A*. *phagocytophilum*, and 20 out of 403 rodent spleens (around ~5%) were tested positive for *A*. *phagocytophilum* in species-specific PCR assays. In the present study, nine out of 47 *A*. *agrarius* (19.1%) were positive for *A*. *phagocytophilum*. Moreover, six out of 45 spleen samples, four out of 46 blood samples, and one out of 47 kidney samples were positive for *A*. *phagocytophilum*. These results displayed 99% similarities with that of the isolates obtained from humans and dogs in ROK (Fig 1A and 1B). This suggests the possibility that *A*. *agrarius* may be the reservoirs of *A*. *phagocytophilum* in ROK.

*L*. *interrogans* causes leptospirosis in both humans and animals. The rodents asymptomatically carry the bacteria in their kidneys and excrete them in urine, thus contaminating the environment [33, 34]. *L*. *interrogans* was detected in only 6.4% of the kidney samples in this study. In addition, PHA results indicate that only one out of 46 (2.2%) blood samples and only one of the 3 PCR-positive kidney samples were serologically positive for *Leptospira* species. This suggests that the kidneys of *A*. *agrarius* may be the main reservoirs of *L*. *interrogans*. From the results of phylogenetic tree analysis, the positive samples demonstrated 99–100% similarity with serovar *lai* isolated from sewage in China and serovar *manilae* isolated from mouse kidney in Japan (Fig 1C–1E). Since 1984, *L*. *interrogans* isolates identified from *A*. *agrarius* field rodents were classified under serogroup Icterohaemorrhagiea as serovar *lai* and *hongchon* in ROK [35].

Our results demonstrated the rate of rodent-borne bacterial infections in different tissues of 47 *A*. *agrarius* collected from June to August 2016 in Gwangju City of ROK. A total of 12 (25.5%) mice were positive for *A*. *phagocytophilum* and *L*. *interrogans* as determined through PCR, and eight (17.4%) sera samples were seropositive as determined by IFA and PHA. *A*. *phagocytophilum* and *L*. *interrogans* were detected in *A*. *agrarius* living in close proximity to humans in the suburban areas, such as Gwangju City. Considering the tissue detection levels of *A*. *phagocytophilum*, the spleen was found to be a more sensitive organ to detect the presence of the pathogen. In contrast, the kidneys were more sensitive to detect the presence of *L*. *interrogans*. All the blood samples were found to be negative for the pathogens by PCR. This signifies that the overall rates of PCR detection can be lower if only blood samples were tested.

The serological assay of *A*. *phagocytophilum* was not performed in this study due to the lack of an antigen slide for *A*. *phagocytophilum*. The data were limited as only a few samples were obtained from only two sites. Future studies, using larger samples collected over a longer period of time, may lead to a better understanding of the nature and spread of these infectious pathogens. Further research is required to monitor bacterial prevalence rates in wild rodents, which serve as reservoir hosts for many infectious pathogens.

In conclusion, the present study indicates that rodent-borne bacterial infections circulating in wild rodent populations may be prevalent in the metropolitan suburban areas of ROK. In particular, the spleen was found to be more sensitive to detect the presence of *A*. *phagocytophilum*, whereas the kidneys were more sensitive for *L*. *interrogans*.

## Supporting information

S1 Table Number of positive bacterial infection among the 47 *Apodemus agrarius* rodents through serological assays and polymerase chain reaction. (DOCX)

S1 File Raw data. (ZIP)
