# Supplementary material for: Prevalence of Orientia tsutsugamushi, Anaplasma phagocytophilum and Leptospira interrogans in striped field mice in Gwangju, Republic of Korea
Source: PLoS One. 2019 Aug 16;14(8):e0215526. doi: 10.1371/journal.pone.0215526 (PMC6697328; doi:10.1371/journal.pone.0215526)
Supplement: S1 Table — (DOCX) [file pone.0215526.s001.docx]

**S1 Table. Number of positive bacterial infection among the 47 *Apodemus agrarius* rodents through serological assays and polymerase chain reaction.**

|  | | ST | | | | Anaplasmosis | | Leptospirosis | | |  | ST | | | Anaplasmosis | | Leptospirosis | |  | ST | | | Anaplasmosis | | Leptospirosis | | |
| --- | --- | --- | --- | --- | --- | --- | --- | --- | --- | --- | --- | --- | --- | --- | --- | --- | --- | --- | --- | --- | --- | --- | --- | --- | --- | --- | --- |
| Name | specimen | *rrs*^a^ | Kit^b^ | 56 kDa^c^ | IFA^d^ | *groEL^e^* | *ankA^f^* | *rpoB^g^* | *LipL32*^h^ | PHA^i^ | Specimen | *rrs* | Kit | 56 kDa | *groEL* | *ankA* | *rpoB* | *LipL32* | Specimen | *rrs* | Kit | 56 kDa | *groEL* | *ankA* | *rpoB* | *LipL32* | gyrB^j^ |
| 6-1 | B | –^k^ | – | – | – | – | **+**^l^ | – | – | – | S | – | – | – | – | **+** | – | – | K | – | – | – | – | – | – | – | – |
| 6-2 | B | – | – | – | – | – | – | – | – | – | S | – | – | – | – | – | – | – | K | – | – | – | – | – | – | – | – |
| 6-3 | B | – | – | – | – | – | – | – | – | – | S | – | – | – | – | – | – | – | K | – | – | – | – | – | – | – | – |
| 6-4 | B | – | – | – | – | – | – | – | – | – | S | – | – | – | – | – | – | – | K | – | – | – | – | – | – | – | – |
| 6-5 | B | – | – | – | – | – | – | – | – | – | S | – | – | – | – | – | – | – | K | – | – | – | – | – | – | – | – |
| 6-6 | B | – | – | – | **1:128** | **+** | **+** | – | – | – | S | – | – | – | – | – | – | – | K | – | – | – | – | – | – | – | – |
| 6-7 | B | – | – | – | **1:64** | – | **+** | – | – | – | S | – | – | – | – | – | – | – | K | – | – | – | – | – | – | – | – |
| 6-8 | B | – | – | – | – | – | – | – | – | – | S | – | – | – | – | – | – | – | K | – | – | – | – | – | – | – | – |
| 6-9 | B | – | – | – | – | – | – | – | – | – | S | – | – | – | – | – | – | – | K | – | – | – | – | – | – | – | – |
| 6-10 | B | – | – | – | – | – | – | – | – | – | S | – | – | – | – | – | – | – | K | – | – | – | – | – | – | – | – |
| 6-11 | B | – | – | – | – | – | – | – | – | – | S | – | – | – | – | – | – | – | K | – | – | – | – | – | – | – | – |
| 6-12 | B | – | – | – | – | – | – | – | – | – | S | – | – | – | – | – | – | – | K | – | – | – | – | – | – | – | – |
| 6-13 | B | – | – | – | – | – | – | – | – | – | S | – | – | – | – | – | – | – | K | – | – | – | – | – | – | – | – |
| 6-14 | B | – | – | – | – | – | – | – | – | – | S | – | – | – | – | – | – | – | K | – | – | – | – | – | – | – | – |
| 6-15 | B | – | – | – | – | – | – | – | – | – | S | – | – | – | – | – | – | – | K | – | – | – | – | – | – | – | – |
| 6-16 | B | – | – | – | – | – | – | – | – | – | S | – | – | – | – | – | – | – | K | – | – | – | – | – | – | – | – |
| 7-1 | B | – | – | – | – | – | – | – | – | – | S | – | – | – | – | – | – | – | K | – | – | – | – | – | – | – | – |
| 7-2 | B | – | – | – | – | – | – | – | – | – | S | – | – | – | – | – | – | – | K | – | – | – | – | – | – | – | – |
| 7-3 | B | – | – | – | – | – | – | – | – | – | S | – | – | – | – | – | – | – | K | – | – | – | – | – | – | – | – |
| 7-4 | B | – | – | – | **1:16** | – | – | – | – | – | S | – | – | – | – | – | – | – | K | – | – | – | – | – | – | – | – |
| 7-5 | B | – | – | – | – | – | – | – | – | – | S | – | – | – | – | – | – | – | K | – | – | – | – | – | – | – | – |
| 7-6 | B | – | – | – | – | – | – | – | – | – | S | – | – | – | – | – | – | – | K | – | – | – | – | – | – | – | – |
| 7-7 | B | – | – | – | – | – | – | – | – | – | S | – | – | – | – | – | – | – | K | – | – | – | – | – | – | – | – |
| 7-8 | B | – | – | – | – | – | – | – | – | – | S | NA^m^ | NA | NA | NA | NA | NA | NA | K | – | – | – | – | – | – | – | – |
| 7-9 | B | – | – | – | – | – | – | – | – | – | S | – | – | – | – | – | – | – | K | – | – | – | – | – | – | – | – |
| 7-10 | B | NA | NA | NA | NA | NA | NA | NA | NA | NA | S | – | – | – | – | – | – | – | K | – | – | – | – | – | – | – | – |
| 7-11 | B | – | – | – | – | – | – | – | – | – | S | – | – | – | – | – | – | – | K | – | – | – | – | – | – | – | – |
| 7-12 | B | – | – | – | – | – | – | – | – | – | S | – | – | – | – | – | – | – | K | – | – | – | – | – | – | – | – |
| 7-13 | B | – | – | – | **1:16** | – | – | – | – | – | S | – | – | – | – | – | – | – | K | – | – | – | – | – | – | – | – |
| 7-14 | B | – | – | – | – | – | – | – | – | – | S | – | – | – | – | – | – | – | K | – | – | – | – | – | – | – | – |
| 7-15 | B | – | – | – | – | – | – | – | – | – | S | – | – | – | – | – | – | – | K | – | – | – | – | – | – | – | – |
| 7-16 | B | – | – | – | – | – | – | – | – | – | S | – | – | – | – | – | – | – | K | – | – | – | – | – | – | – | – |
| 7-17 | B | – | – | – | **1:16** | – | – | – | – | **1:160** | S | – | – | – | – | – | – | – | K | – | – | – | – | – | **+** | **+** | **+** |
| 7-18 | B | – | – | – | **1:32** | – | – | – | – | – | S | – | – | – | – | – | – | – | K | – | – | – | – | – | **+** | **+** | **+** |
| 8-1 | B | – | – | – | – | – | – | – | – | – | S | – | – | – | – | **+** | – | – | K | – | – | – | – | – | – | – | – |
| 8-2 | B | – | – | – | – | **+** | **+** | – | – | – | S | – | – | – | – | **+** | – | – | K | – | – | – | – | – | – | – | – |
| 8-3 | B | – | – | – | – | **+** | – | – | – | – | S | – | – | – | – | – | – | – | K | – | – | – | – | – | – | – | – |
| 8-4 | B | – | – | – | – | – | – | – | – | – | S | – | – | – | – | – | – | – | K | – | – | – | – | – | – | – | – |
| 8-5 | B | – | – | – | – | – | – | – | – | – | S | – | – | – | – | – | – | – | K | – | – | – | – | – | – | – | – |
| 8-6 | B | – | – | – | – | – | – | – | – | – | S | – | – | – | – | **+** | – | – | K | – | – | – | – | **+** | – | – | – |
| 8-7 | B | – | – | – | **1:256** | – | – | – | – | – | S | – | – | – | – | – | – | – | K | – | – | – | – | – | – | – | – |
| 8-8 | B | – | – | – | – | – | – | – | – | – | S | – | – | – | – | – | – | – | K | – | – | – | – | – | – | – | – |
| 8-9 | B | – | – | – | **1:64** | – | – | – | – | – | S | NA | NA | NA | NA | NA | NA | NA | K | – | – | – | – | – | **+** | – | – |
| 8-10 | B | – | – | – | – | – | – | – | – | – | S | – | – | – | – | **+** | – | – | K | – | – | – | – | – | – | – | – |
| 8-11 | B | – | – | – | – | – | – | – | – | – | S | – | – | – | – | – | – | – | K | – | – | – | – | – | – | – | – |
| 8-12 | B | – | – | – | – | **+** | – | – | – | – | S | – | – | – | – | **+** | – | – | K | – | – | – | – | – | – | – | – |
| 8-13 | B | – | – | – | – | – | – | – | – | – | S | – | – | – | – | – | – | – | K | – | – | – | – | – | – | – | – |
| No. total samples | **46** | 0 | 0 | 0 | **8** | **4** | **4** | 0 | 0 | **1** | **45** | 0 | 0 | 0 | 0 | **6** | 0 | 0 | **47** | 0 | 0 | 0 | 0 | **1** | **3** | **2** | **2** |

^a^ 16S ribosomal RNA

^b^ INNOPLEX TSUTSU detection kit for *O. tsutsugamushi*

^c^ 56 kDa gene

^d^ cut off for Immunofluorescence Assay, Immunoglobulin G ≥ 1:16

^e^ Heat shock protein chaperone

^f^ Ankyrin-related protein gene

^g^ RNase polymerase subunit beta

^h^ Outer membrane lipoprotein

^i^ cut off for Passive Hemagglutination Assay ≥ 1:80

^j^ DNA gyrase subunit B

^k^ –: negative

^l^ +: positive

^m^ NA: not available

ST; scrub typhus, B; blood, S; spleen, K; kidney
