# Supplementary figures and images for: Prevalence of Orientia tsutsugamushi, Anaplasma phagocytophilum and Leptospira interrogans in striped field mice in Gwangju, Republic of Korea
Source: PLoS One. 2019 Aug 16;14(8):e0215526. doi: 10.1371/journal.pone.0215526 (PMC6697328; doi:10.1371/journal.pone.0215526)

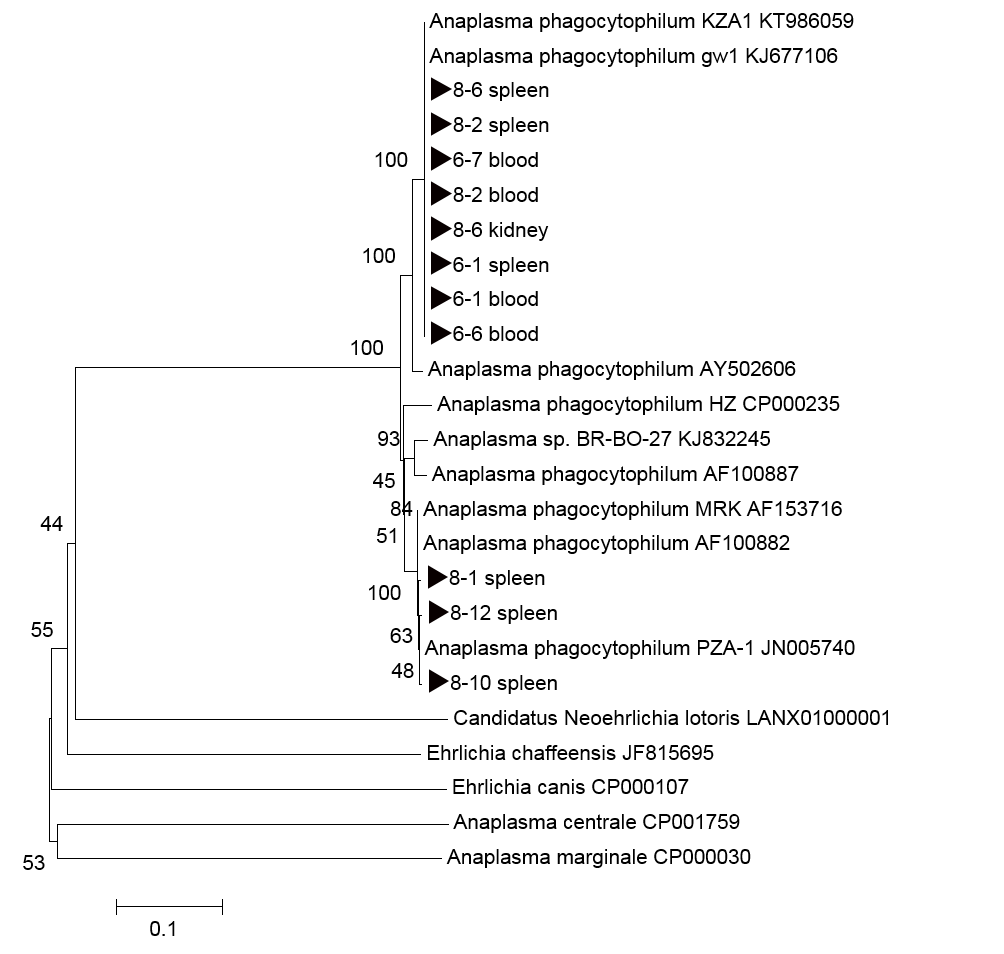

Supplement: S1 File — (ZIP) [file pone.0215526.s002.zip › Supplement_wild rodent_2016/Fig 1/Anaplasma ankA (560bp) wild rodent 2016.tif]

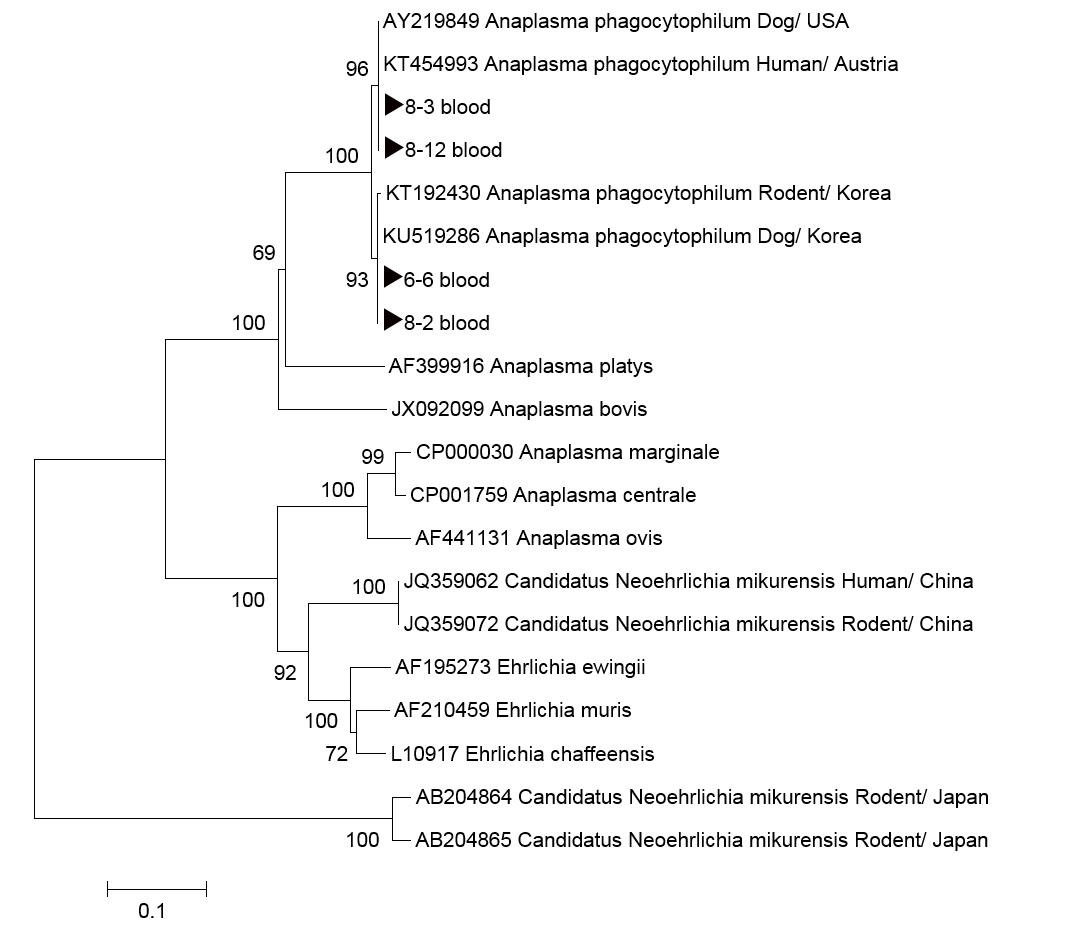

Supplement: S1 File — (ZIP) [file pone.0215526.s002.zip › Supplement_wild rodent_2016/Fig 1/Anaplasma groEL (330bp) wild rodent 2016.tif]

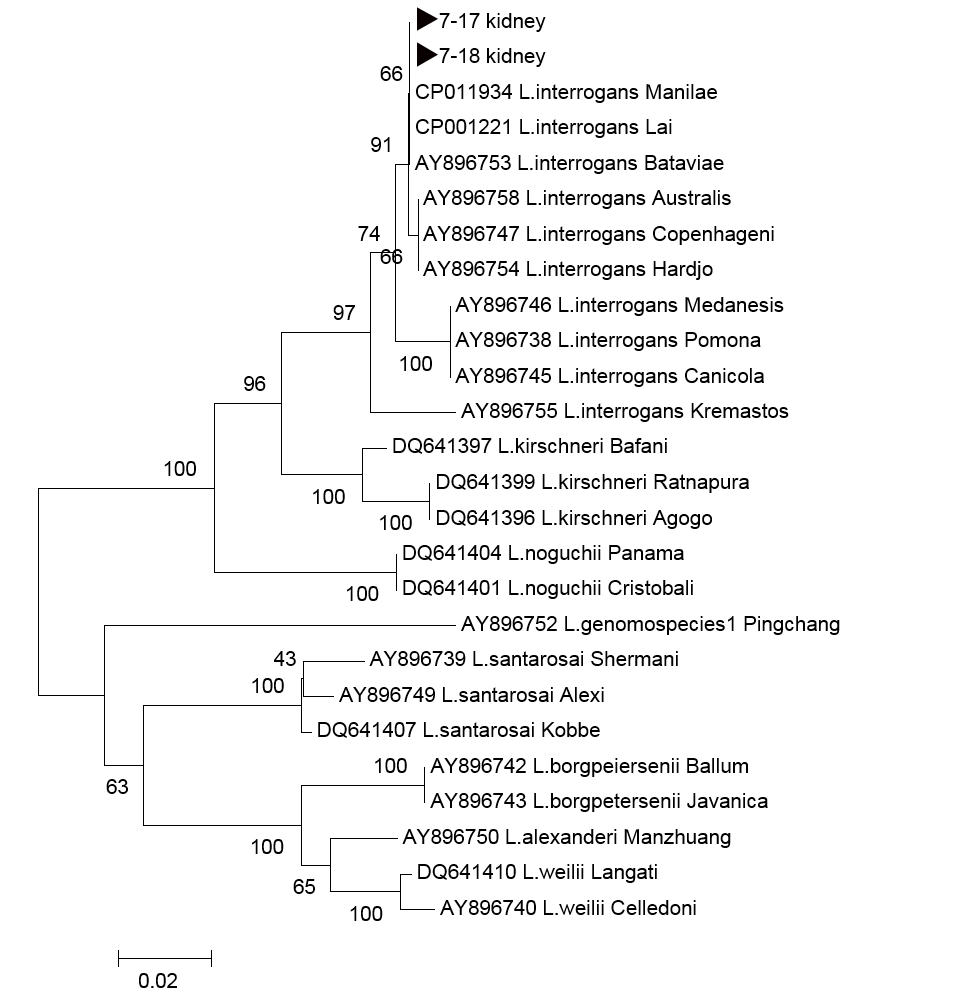

Supplement: S1 File — (ZIP) [file pone.0215526.s002.zip › Supplement_wild rodent_2016/Fig 1/Leptospira gyrB (400bp) wild rodent 2016.tif]

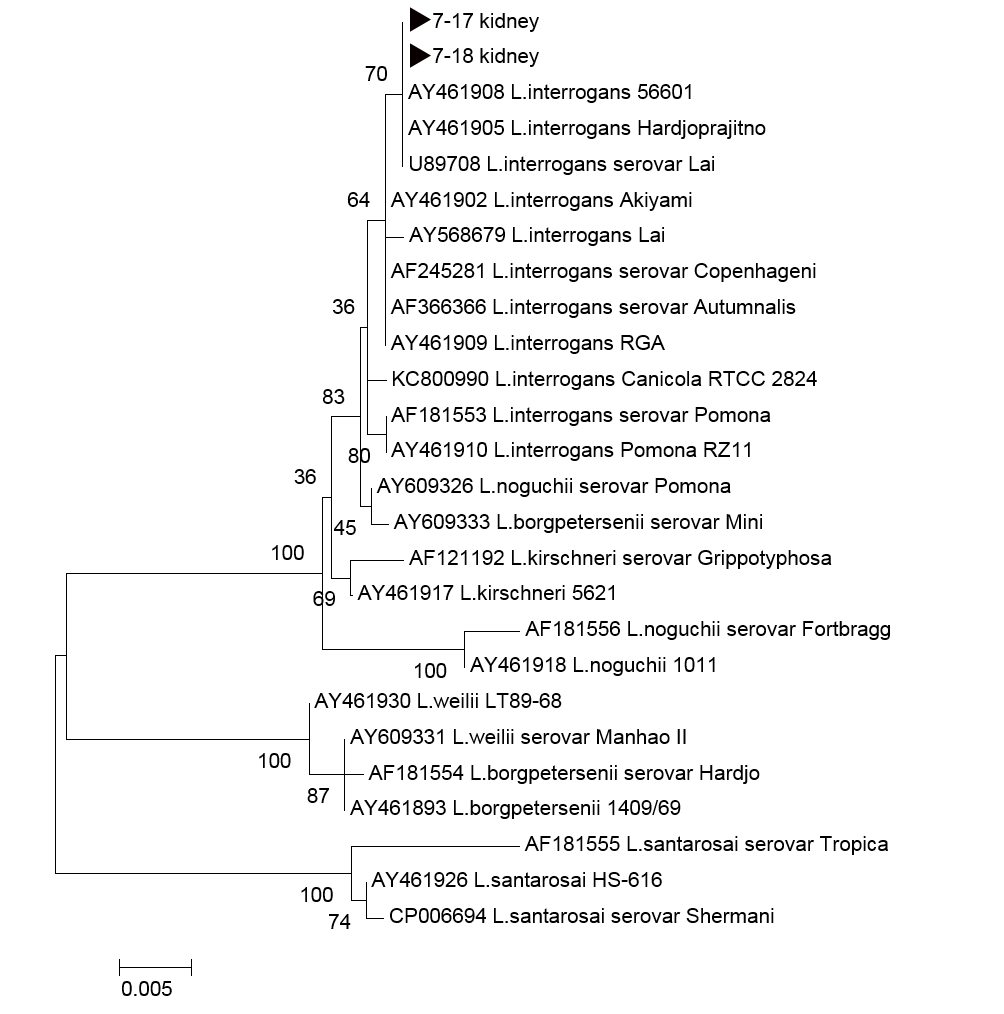

Supplement: S1 File — (ZIP) [file pone.0215526.s002.zip › Supplement_wild rodent_2016/Fig 1/Leptospira LipL32 (780bp) wild rodent 2016.tif]

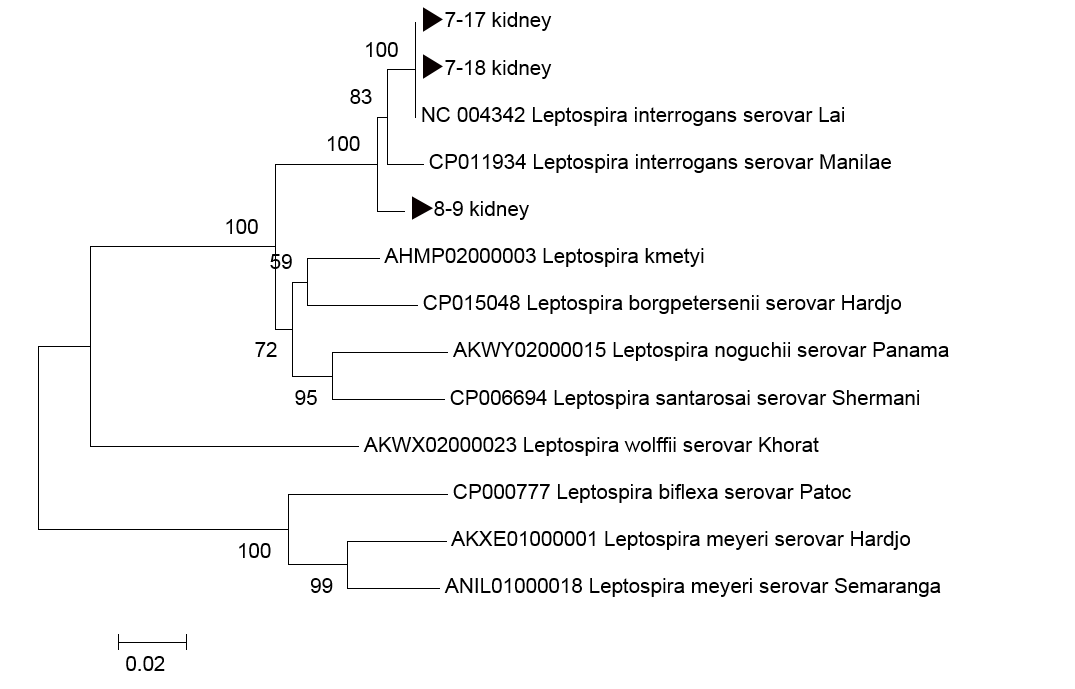

Supplement: S1 File — (ZIP) [file pone.0215526.s002.zip › Supplement_wild rodent_2016/Fig 1/Leptospira rpoB (890bp) wild rodent 2016.tif]
